# Evaluating the antiviral efficacy and specificity of chlorogenic acid and related herbal extracts against SARS-CoV-2 variants via spike protein binding intervention

**DOI:** 10.1016/j.jtcme.2024.11.009

**Published:** 2024-11-19

**Authors:** Wen-Yu Hsieh, Chu-Nien Yu, Chang-Chang Chen, Chun-Tang Chiou, Brian D. Green, Oscar K. Lee, Chia-Chune Wu, Ly Hien Doan, Chi-Ying F. Huang, Cheng Huang, Chien-Ju Liu, Yu-Hsin Chen, Jing-Jy Cheng, Heng-Chih Pan, Hui-Kang Liu

**Affiliations:** a Biomedical Industry Ph.D. Program, National Yang Ming Chiao Tung University, Taipei, 11221, Taiwan; b Institute of Clinical Medicine, National Yang Ming Chiao Tung University, Taipei, 11221, Taiwan; c Division of Chinese Materia Medica Development, National Research Institute of Chinese Medicine, Ministry of Health and Welfare, Taipei, 11221, Taiwan; d Institute for Global Food Security, School of Biological Sciences, Queen's University Belfast, Belfast, BT7 1NN, UK; e Department of Orthopedics, China Medical University Hospital, Taichung, Taiwan; f Institute of Biopharmaceutical Sciences, College of Pharmaceutical Sciences, National Yang Ming Chiao Tung University, Taipei, 11221, Taiwan; g Institute of Biotechnology, Vietnam Academy of Science and Technology, Hanoi, Viet Nam; h Program in Molecular Medicine, College of Life Sciences, National Yang Ming Chiao Tung University, Taipei, 11221, Taiwan; i Taiwan National Graduate Program in Molecular Medicine, Academia Sinica, National Yang Ming Chiao Tung University, Taipei, 11221, Taiwan; j Institute of Clinical Medicine, College of Medicine, National Yang Ming Chiao Tung, Taipei, 11221, Taiwan; k Department of Biotechnology and Laboratory Science in Medicine, School of Biomedical Science and Engineering, National Yang Ming Chiao Tung, Taipei, 11221, Taiwan; l Department of Biochemistry, School of Medicine, Kaohsiung Medical University, Kaohsiung, 80708, Taiwan; m Department of Earth and Life Sciences, University of Taipei, Taipei, 11036, Taiwan; n Tea and Beverage Research Station, Ministry of Agriculture, Yangmei Township, Taoyuan County, 326011, Taiwan; o Taichung District Agricultural Research and Extension Station, Ministry of Agriculture, Chang-Hwa County, 515008, Taiwan; p Division of Basic Chinese Medicine, National Research Institute of Chinese Medicine, Ministry of Health and Welfare, Taipei, 11221, Taiwan; q Department of Nephrology, Chang Gung Memorial Hospital, Keelung City, 204201, Taiwan; r Ph.D. Program in the Clinical Drug Development of Herbal Medicine, Taipei Medical University, Taipei, 11031, Taiwan; s Traditional Herbal Medicine Research Center, Taipei Medical University Hospital, Taipei, 11031, Taiwan

**Keywords:** COVID-19, SARS-CoV-2 variants, Spike protein, Chlorogenic acid, Green coffee bean, *Echinacea purpurea*, H1N1 influenza virus

## Abstract

Severe acute respiratory syndrome coronavirus 2 (SARS-CoV-2) and influenza A virus infect the respiratory tract through surface proteins, causing similar symptoms. Since the onset of the COVID-19 pandemic, both viruses have posed significant ongoing global health threats. Like the influenza virus, SARS-CoV-2 evolves into variants that can reduce vaccine efficacy. Thus, herbal medicines are being explored as supplementary options to enhance protection against these infections. This study aimed to investigate the therapeutic potential of chlorogenic acid (3-CQA) and related extracts from green coffee beans and *Echinacea purpurea* against SARS-CoV-2 variants and H1N1 infection. The methods employed included an ELISA-based trimeric spike protein binding assay, viral infection assays, plaque assays, and molecular docking studies. Results showed that 3-CQA blocked spike protein/angiotensin converting enzyme 2 (ACE2) binding for most SARS-CoV-2 variants of concern, except the Delta variant. Extracts from Green coffee bean and *E. purpurea* effectively blocked all variants tested. Additionally, antibodies blocked spike protein binding up to Omicron BA.2. Molecular docking suggested that 3-CQA binding to Omicron BA.1, BA.2, and BA.4, though not to the Delta spike protein, may lead to steric hindrance, preventing receptor-binding domain interactions with ACE2. Finally, both 3-CQA and *E. purpurea* extract showed preventive effects against H1N1 viral infection, though with lower potency compared to SARS-CoV-2. In conclusion, 3-CQA has potential as a phytoconstituent marker for herbs with bioactive properties against SARS-CoV-2 and H1N1 viral infections.

## Graphical abstract

**Figure:**
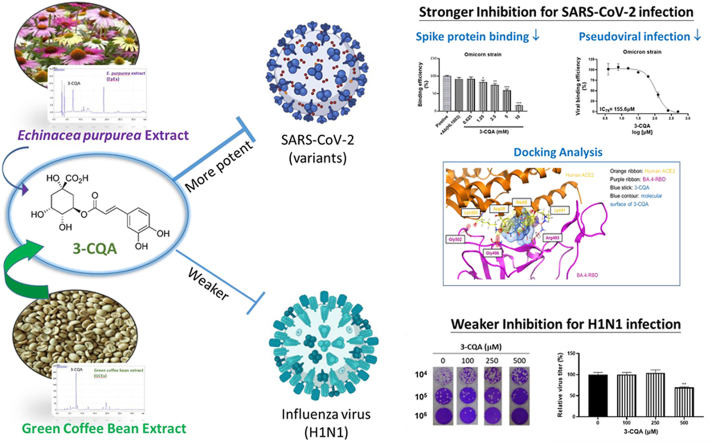

## 1 Introduction

Severe acute respiratory syndrome coronavirus 2 (SARS-CoV-2) and influenza A virus both infect the respiratory tract via surface proteins, causing similar symptoms. Influenza, commonly known as the flu, seasonally affects up to 1 billion people worldwide and results in approximately half a million deaths annually due to respiratory complications.^1^ In contrast, as of July 2024, the World Health Organization (WHO) reports that more than 775 million people globally have suffered from coronavirus disease 2019 (COVID-19), caused by SARS-CoV-2 infection, with over 7 million deaths since the virus was first identified in December 2019.^2^ Acute respiratory distress syndrome (ADRS) is a severe complication linked to virus-induced “cytokine storms” and the development of pulmonary fibrosis.^3^ A proportion of survivors have reported ‘long COVID’ symptoms, even in cases without hospitalization.^4^ Persistent SARS-CoV-2 spike protein in the blood has been associated with these post-acute sequelae of COVID-19 (PASC).^5^

Antibody-based therapeutics, including neutralizing antibodies, COVID-19 convalescent sera, vaccines (mRNA, inactivated, viral-vector based), are regarded as the primary prevention and treatment strategies against SARS-CoV-2. As of August 2022, approximately 12 billion vaccine doses have been administered globally.^2^^,^^6^

Like other coronaviruses, SARS-CoV-2 mediated host cell entry primarily depends on the binding of the spike protein to the ACE2 receptor.^7^ The receptor-binding domain (RBD) of the spike protein plays a crucial role in initiating complex formation with ACE2, which facilitates subsequent membrane fusion.^8^ Disruption of RBD-ACE2 complex formation is a major target in therapeutic antibody development. Unfortunately, since the identification of the reference strain in Wuhan, China, mutations have led to multiple variants of concern (VOC), from Alpha to Omicron, which have reduced vaccine effectiveness.^9^^,^^10^ In response to emerging variants, pharmaceutical companies have continued developing next-generation vaccines as boosters.^11^ However, reports of reinfections and vaccine inequity in low-income countries remain significant public health challenges.^12^^,^^13^

Regarding non-vaccine treatments for SARS-CoV-2, by September 2021, over 640 drugs were in development, with 470 clinical trials reviewed by the FDA, and 11 treatments authorized for emergency use, including one FDA-approved treatment.^14^ Potential molecular targets for anti-SARS-CoV-2 therapies include viral spike proteins, human angiotensin-converting enzyme 2 (ACE2), transmembrane serine protease 2 (TMPRSS2), RNA-dependent RNA polymerase (RdRp), and SARS-CoV-2 M^pro 15^. Herbal medicines and dietary supplements have also played a supportive role in managing the COVID-19 pandemic, utilizing mechanisms similar to those of non-vaccine treatments.^16^, ^17^, ^18^, ^19^ Among these mechanisms, blocking the spike protein's binding to ACE2 with various phytochemicals could directly inhibit viral entry into host cells.^20^, ^21^, ^22^ Chlorogenic acid (3-CQA), the most abundant isomer among caffeoylquinic acids, has been proposed as a potential SARS-CoV-2 therapeutic.^23^^,^^24^ Chlorogenic acids are also abundant in many foods and herbs associated with SARS-CoV-2 intervention and the management of COVID-19^15^^,^^25^, ^26^, ^27^. However, previous studies have only demonstrated 3-CQA's inhibition of spike protein binding from the reference strain,^25^^,^^26^ with limited data on 3-CQA's effectiveness against SARS-CoV-2 variants or other respiratory viruses such as the H1N1 influenza virus.

Therefore, this study employed multiple platforms to investigate the protective spectrum and specificity of 3-CQA against SARS-CoV-2 variants of concern. Additionally, we selected two herbal extracts—green coffee bean extract (GCEx) and Echinacea purpurea extract (EpEx)—with differing 3-CQA levels. *Echinacea purpurea* is known for its ethnopharmacological applications in treating coughs, chest conditions, sore throat, colds, and influenza,^28^^,^^29^ whereas green coffee beans are less commonly used for these purposes.

## 2 Materials and methods

### 2.1 Extraction method and HPLC analysis

Green coffee bean from TRES, Taiwan and *E. purpurea* shoot from TDARES, Taiwan were milled and extracted with 95 % EtOH (1:20, sample: solvent, w/v) three times at 50 °C. For HPLC analysis, 1 mg/mL green coffee bean extract (GCEx) was made with MeOH and 1 mg/mL of *Echinacea* shoot extract (EpEx) was made with 80 % MeOH. Both samples were filtered through a 0.22 μm Millex®-GV syringe filter (13 mm) (Merck Millipore Ltd.) into 2 mL HPLC vails. HPLC analysis was performed according to the method of He et al. with modification.^30^ HPLC system (Shimadzu CBM-20A communications bus module) equipped with a Shimadzu SPD-M20A diode array detector (DAD), Shimadzu LC-40DXR Pump, Shimadzu SIL-40CXR auto sampler was used for analysis. Samples were separated on Shimadzu CTO-40S column oven and an Atlantis® T3 column (Waters Corporation, 5 μm, 4.6 mm × 250 mm) using the following gradient condition: 15 % A in 0–3 min, 15%–25 % A in 3–15 min, 25–35 % A in 15–35 min and 15 % A in 35.01–40 min at a flow rate of 1 mL/min with 27 °C, 10 μL of injection volume, and PDA detection at 300 nm. All solvents were HPLC grade. Solvent A was acetonitrile, and solvent B was 0.05 % phosphoric acid. 1 mg/L of chlorogenic acid standard (ChromaDex Inc.) in MeOH was prepared and diluted into a concentration gradient (0.8–100 mg/L) for plotting the standard curve. The R^2^ of standard curve was 0.9998.

### 2.2 ELISA based spike protein binding assay

Enzyme-linked immunosorbent assay (ELISA) based spike protein binding assay employed trimeric SARS-CoV-2 Spike proteins from reference strain (Wuhan strain) or variant of concern (α, β, γ, δ, ο variants) to interact with biotinylated human ACE2 recombinant protein according to the procedures previously described.^15^

### 2.3 Cell culture

Human embryonic kidney (HEK-293T/17; ATCC® CRL-11268TM) cells were obtained from the American Type Culture Collection (ATCC) and used to generate a stable cell line (HEK-293T-ACE2) expressing human ACE2 protein by transducing VSV-G pseudotyped lentivirus carrying human ACE2 gene. HEK-293T-ACE2 cells were cultured with Dulbecco's Modified Eagle Medium (DMEM; Gibco) containing 10 % (v/v) fetal bovine serum (Gibco), 1x penicillin-streptomycin (Gibco), and 2.5 μg/ml puromycin at 37 °C. Mammalian Madin-Darby canine kidney (MDCK; ATCC® CCL-34) cells can be infected by influenza virus and are commonly used for influenza vaccine productions.^31^ MDCK cells were cultured with Eagle's Minimum Essential Medium (EMEM; Gibco) containing 10 % (v/v) fetal bovine serum (Gibco), 1x penicillin-streptomycin (Gibco), and 2.5 μg/ml puromycin at 37 °C.

### 2.4 Viral infection efficiency analysis

SARS-CoV-2 Spike Pseudotyped Lentivirus was produced by the National RNAi Core Facility at Academia Sinica, Taiwan. This virus can infect human ACE2 expressing cells or human ACE2 transgenic mice (Supplementary Fig. 1). To study effect of drugs on viral infection efficiency, the synthetic DNA fragment encoding SARS-CoV-2 Spike gene(s) including BA.1, BA.2, and BA.4/5 variants was purchased from Integrated DNA Technologies (IDT, Coralville, IA, USA) and cloned into the *Kpn*I and *Eco*R*I* restriction enzyme sites of pcDNA™3.1 (+) mammalian expression vector (sTable 1; Thermo Fisher Scientific Inc., Waltham, MA USA).

The pseudotyped lentivirus carrying SARS-CoV-2 spike protein was generated by transiently transfecting HEK-293T cells with pCMV-ΔR8.91, pLAS2w.Fluc.Ppuro and pcDNA3.1-nCoV-S (BA.1, BA.2, and BA.4/5). Viral particles were harvested 48h post-transfection. Cell debris was removed by centrifugation at 4000×*g* for 10 min, and the supernatant was filtered with 0.45-μm syringe filter (Pall Corporation) prior to being stored at −80 °C.

For the viral infection efficiency analysis, equal volume of heat-inactivated sera was used for preparing serially diluted test agents with desired dilution and incubated with the volume of 1000 TU of SARS-CoV-2 pseudotyped lentivirus in DMEM (supplemented with 1 % FBS and 100 U/ml Penicillin/Streptomycin) for 1 h at 37 °C. The mixture was then inoculated with HEK-293T-ACE2 cells at 10,000 cells per well in 96-well plates. The culture medium was replaced with fresh complete DMEM (supplemented with 10 % FBS and 100 U/ml Penicillin/Streptomycin) at 16h post-infection and cells were continuously cultured for another 48h before performing luciferase assay. For performing luciferase assay, the luciferase activity was determined by using Bright-Glo™ Luciferase Assay System (Promega, Madison, WI, USA). The relative light unit (RLU) of was detected by Molecular Devices-SpectraMaxL (Molecular Devices Inc., Sunnyvale, CA, USA). The percentage of virus infection was calculated by using the difference of RLU value between the virus only control and the presence of diluted serum. The calculation formula was shown below:

(RLU ^Control^ - RLU ^Serum^) / RLU ^Control^ x 100 (%)

The potential toxicity of test agent on HEK-293T-ACE2 cells was measured by MTT (3-(4,5-dimethylthiazol-2-yl)-2,5-diphenyltetrazolium bromide; Sigma) assay.

### 2.5 Protein-ligand docking analysis

*In silico* protein-ligand docking of chlorogenic acid (3-CQA) against different SARS-CoV-2 variant spike proteins were performed using Molecular operating environment (MOE) software 2022.02 (Chemical Computing Group ULC, Montreal, QC, Canada).

The 3D structure of chlorogenic acid was downloaded from NCBI PubChem database (PubChem CID: 1794427). The protein structures of SARS-CoV-2 Delta (PDB accession code: 7W9C), Omicron BA.1 (7U0N), BA.2 (7ZF7), and BA.4 (7XNS) spike protein/human ACE2 complexes were downloaded from RCSB protein data bank (PDB).^32^, ^33^, ^34^, ^35^ Chemical and protein structures were imported into MOE software and the pre-bound ligands were first removed from PDB structure. The structure is then prepared by MOE “QuickPrep” tool, including energy minimization by fixing atoms, applying tethers, deleting unnecessary water molecules and performing initial structural refinements.

Conformational database of chlorogenic acid was generated by MOE “Conformational Search” tool. Active sites of SARS-CoV-2 variant spike protein RBD were found by MOE “Site Finder” tool, and active sites near human ACE2 binding site were selected for later molecular docking. Conformational database of chlorogenic acid was docked to SARS-CoV-2 spike protein RBD by MOE “Dock” tool. The docking structure with minimum S score, having the highest binding affinity, was selected for further protein contact analyses. Amino acids involved in protein-ligand interaction, as well as interaction bond type, bond length, and bond energy were indicated by MOE “Protein Contacts” tool. 2D interaction diagram was created by MOE “Ligand Interactions” tool.

For protein superposition analyses, prepared spike RBD/human ACE2 complex structure and docked spike RBD/chlorogenic acid structure were imported into MOE software. The two structures were then aligned and superposed by MOE “Protein Align/Superpose” tool. Molecular surfaces of chlorogenic acid and human ACE2 were generated by MOE “Surfaces and Maps” tool.

### 2.6 Influenza virus culture

The Influenza virus strain A/Brisbane/02/2018 (H1N1) was kindly provided by the Taiwan Centers for Disease Control, Ministry of Health and Welfare, Taiwan. Madin-Darby Canine Kidney (MDCK) cells were purchased from the Bioresource Collection and Research Center, Food Industry Research and Development Institute, Taiwan. MDCK cells were cultured in growth medium (Dulbecco Modified Eagle Medium (DMEM) supplemented with 10 % fetal bovine serum (FBS), 100 units/ml penicillin, and 100 μg/ml streptomycin). All cell culture reagents were obtained from Invitrogen (Grand Island, NY, USA).

### 2.7 Evaluation of anti-influenza activity

MDCK cells were seeded in a 12-well microplate and cultured in growth medium overnight. The influenza virus was pretreated with 3-CQA, EpEx, and GCEx on ice for 1h. The cells were then washed twice with PBS and inoculated with pretreated influenza virus at a multiplicity of infection of 0.1 for 1 h. Afterward, the inoculum was removed, and the cells were cultured in infected medium, DMEM supplemented with 0.2 % bovine serum albumin (Millipore, Billerica, MA, USA) and L-1- tosylamido-2-phenylethyl chloromethyl ketone (TPCK) treated trypsin (Sigma, St. Louis, MO, USA) at 2 μg/ml, for 24 h. The cell medium was collected for a plaque-forming assay to verify the nascent virus titers.

### 2.8 Plaque forming assay

Virus dilution was prepared by performing a 10-fold serial dilution and then infecting 90 % confluent MDCK cells at 37 °C for 1 h. After infection, the cells were overlaid with 1 % Neocel® (Mingtai Chemical, Taoyuan city, Taiwan) in the infection medium. Following a 2-day incubation, the cells were fixed with 10 % formaldehyde and stained with 0.5 % crystal violet.

### 2.9 Statistics

Data are presented as the mean ± standard error of the mean. Statistical analyses were performed using GraphPad Prism 8.0 (GraphPad, San Diego, CA, USA). Multiple group comparison was made by employing ordinary one-way ANOVA with Dunnett's multiple comparisons test. P values less than 0.05 were considered significant.

## 3 Results

**Fig. 1:**
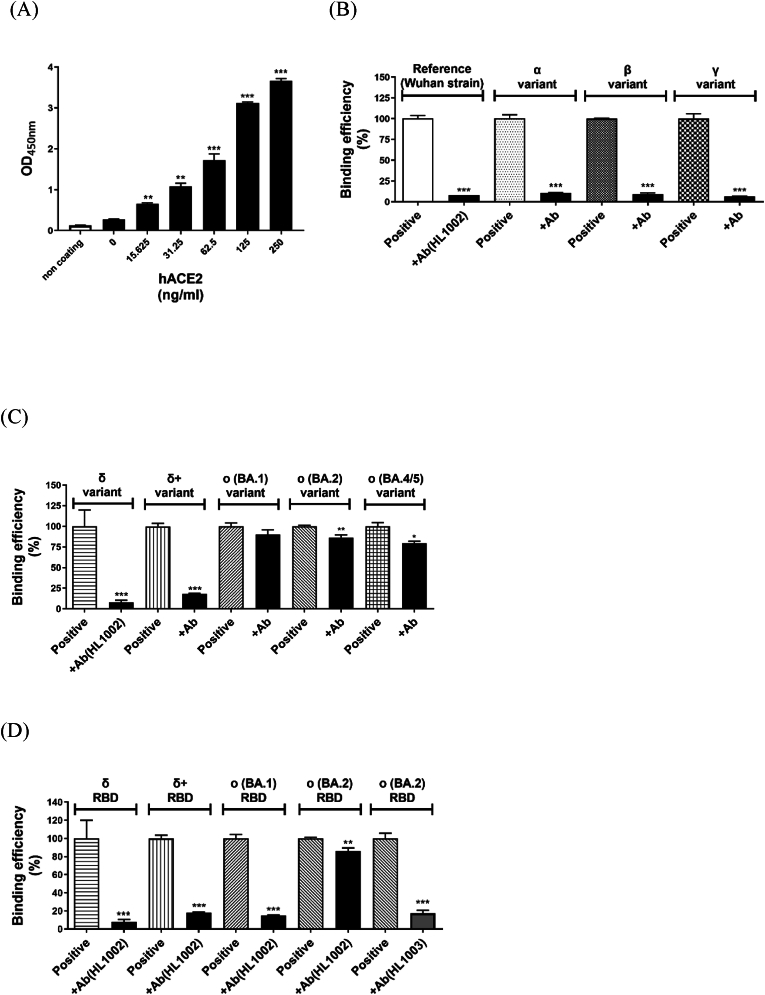
**Effects of RBD monoclonal antibodies on the binding of trimeric spike proteins from SARS-COV-2 variants towards hACE2 protein.** (A) Establishment of spike/hACE2 binding assay. (B) Effects of RBD monoclonal antibody (HL1002) on previous variant of concern (VOC) spike proteins (from α to γ) binding to hACE2. (C) Effects of RBD monoclonal antibody on the current VOC spike proteins (from δ to O) binding to hACE2. (D) Differential effects of RBD monoclonal antibodies on O micron variants. Data are presented as the mean ± SEM (n = 3). ∗P < 0.05, ∗∗P < 0.01, and ∗∗∗P < 0.001 compared with positive control.

Fig. 1A shows concentration-dependent ELISA based protein binding of human ACE2 to the extracellular domain (ECD) of trimeric spike protein. The monoclonal antibody (HL1002; 10 μg/ml) targeting RBD of the reference (Wuhan strain) of SARS-COV-2 virus effectively blocks the binding of trimeric spike protein to hACE2. HL1002 also blocked other variants including α, β, γ, δ, and δ+ variants (Fig. 1B and C). However, blocking effects of HL1002 against Omicron variants (BA.1, BA.2, and BA.4/5) was negligible. It should be noted that BA.4 and BA.5 share the same protein sequence at the RBD. Use of the RBD spike protein restored the blocking effects of HL1002 for the BA.1 variant (Fig. 1D). Unlike HL1003 antibody the blocking effects of HL1002 on BA.2 variant remained poor. Therefore, HL1002 was used as a reference inhibitor against spike proteins from reference strains/variants α, β, γ, and δ, and HL1003 was used for Omicron variants.

**Fig. 2:**
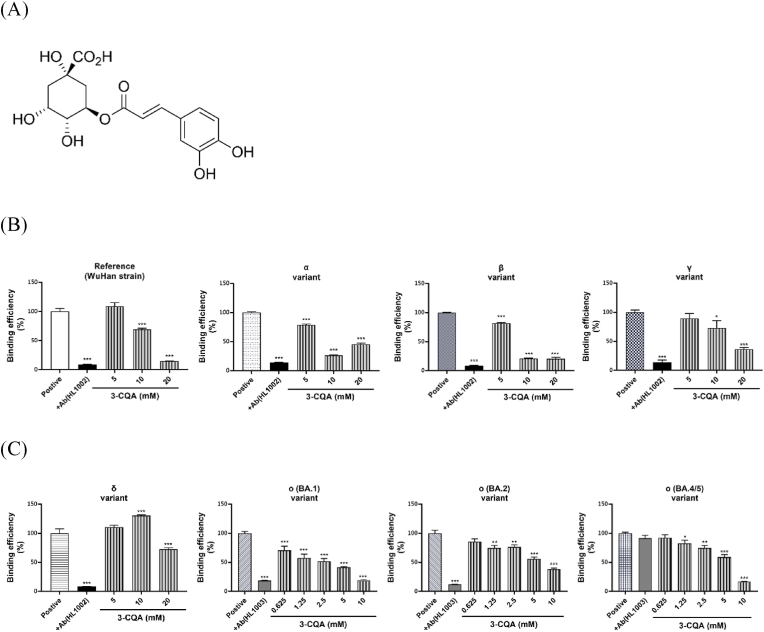
**Effects of chlorogenic acid (3-CQA) on VOC spike proteins binding to hACE2 protein.** (A) Chemical structure of 3-CQA. (B) Effects of 3-CQA on the previous VOC spike proteins (from α to γ) binding to hACE2. (C) Effects of 3-CQA on the current VOC spike proteins (from δ to O) binding to hACE2. Data are presented as the mean ± SEM (n = 3). ∗P < 0.05, ∗∗P < 0.01, and ∗∗∗P < 0.001 compared with positive control.

The inhibitory effects of 3-CQA (Fig. 2A) on previous variants of concern (VOC; Fig. 2B) and current VOC (Fig. 2C) were evaluated. 3-CQA significantly inhibited trimeric spike protein binding of the reference Wuhan strain and was fully effective at 20 mM. For spike proteins from α and β variants 3-CQA inhibited at the concentrations between 5 and 20 mM, and was most effective at 10 mM. The inhibitory effects of 3-CQA for γ and δ variants were comparably weaker and unable to reach the inhibitory activity of HL1002. The inhibitory effect of 3-CQA against Omicron variants BA.1, BA.2, and BA.4/5 was very effective at 10 mM. In contrast, HL1003 was unable to block the trimeric BA.4/5 spike protein binding to human ACE2.

**Fig. 3:**
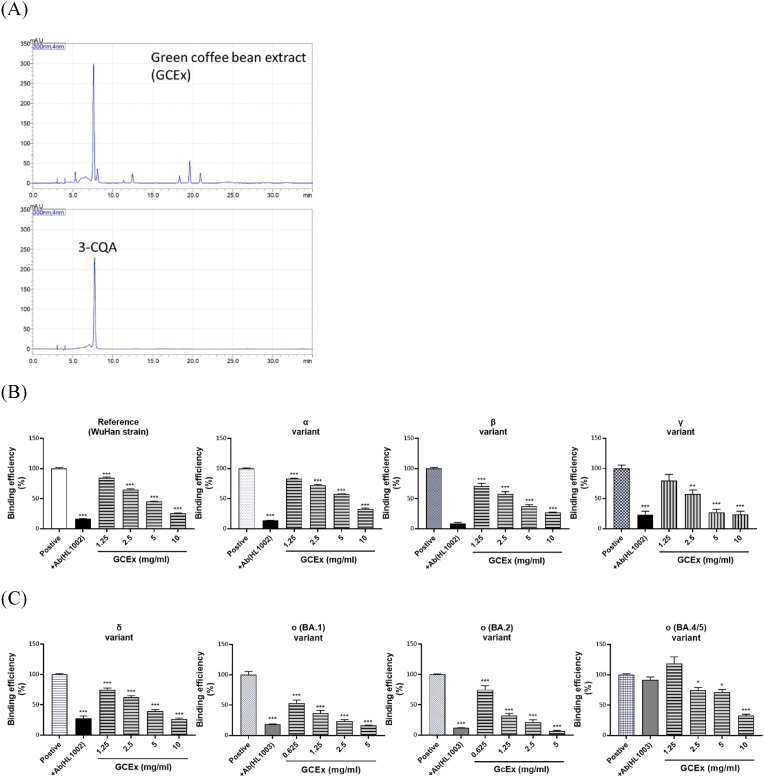
**Effects of Green coffee been extract (GCEx) on VOC spike proteins binding to hACE2 protein.** (A) Identification of 3-CQA in GCEx. (B) Effects of GCEx on the previous VOC spike proteins (from α to γ) binding to hACE2. (C) Effects of GCEx on the current VOC spike proteins (from δ to O) binding to hACE2. Data are presented as the mean ± SEM (n = 3). ∗P < 0.05, ∗∗P < 0.01, and ∗∗∗P < 0.001 compared with positive control.

As shown in Fig. 3A, the chemical fingerprint of GCEx is shown. GCEx is rich in 3-CQA with an estimated content of about 130.9 mg/g 3-CQA appears to be the major constituent in the extract. GCEx effectively blocked the binding of all variant trimeric spike proteins at concentrations between 0.625 and 10 mg/ml as determined by ELISA (Fig. 3B and C). The blocking effect of GCEx was particularly effective when encountering the spike protein from ο (BA.1 and BA.2) variants.

**Fig. 4:**
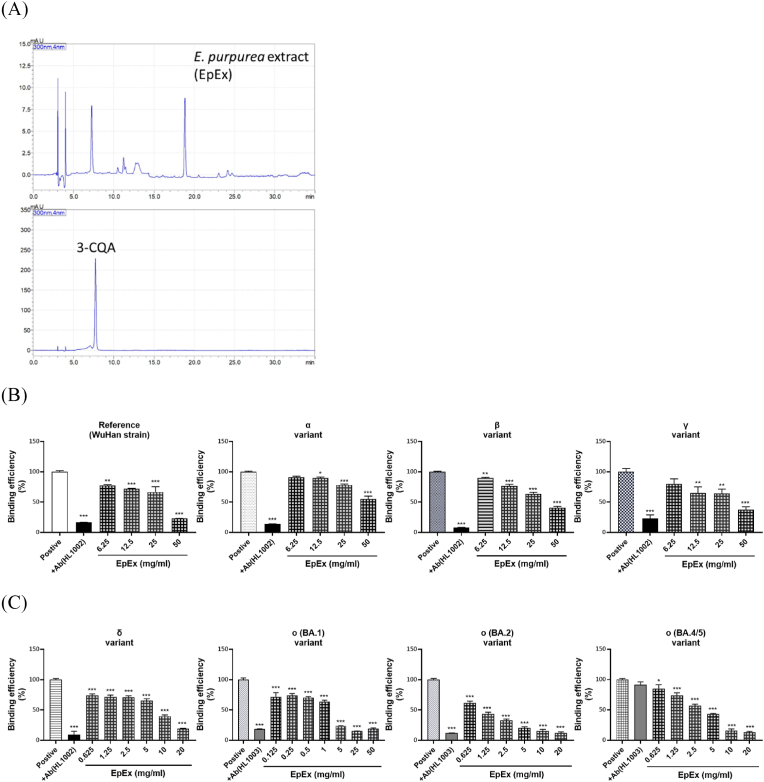
**Effects of *Echinacea purpurea* extract (EpEx) on VOC spike proteins binding to hACE2 protein.** (A) Identification of 3-CQA in EpEx. (B) Effects of EpEx on the previous VOC spike proteins (from α to γ) binding to hACE2. (C) Effects of EpEx on the current VOC spike proteins (from δ to O) binding to hACE2. Data are presented as the mean ± SEM (n = 3). ∗P < 0.05, ∗∗P < 0.01, and ∗∗∗P < 0.001 compared with positive control.

As shown in Fig. 4A, the chemical fingerprint of EpEx is shown. The content of 3-CQA in EpEx is about 4.2 mg/g, which is also one of the major ingredients. ELISA assay results showed that EpEx was effective against all variant trimeric spike proteins at the concentrations between 0.125 and 50 mg/ml (Fig. 4B and C). EpEx was less effective at blocking the spike protein from previous VOC than current VOC. The results were also shown that the blocking effect of EpEx were particularly effective when encountering spike protein from ο (BA.1, BA.2, and BA4/5) variants.

**Fig. 5:**
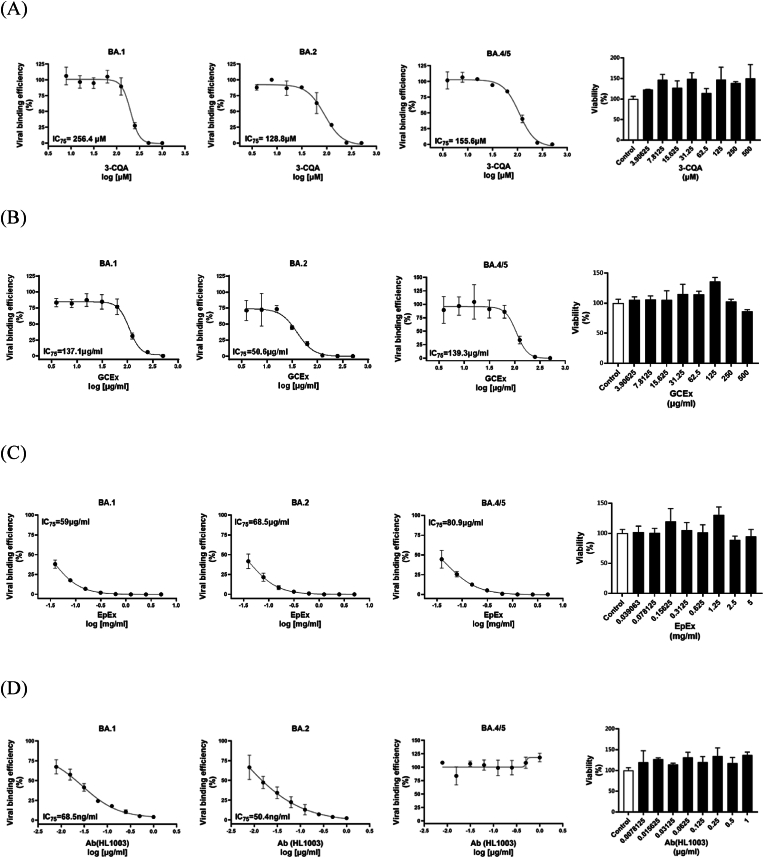
**The ability of 3-CQA, GCEx, EpEx, and antibody to neutralize SARS-CoV-2 Spike Pseudotyped Lentivirus omicron variants.** Anti-viral effects of (A) 3-CQA, (B) GCEx, (C) EpEx, and (D) antibody (HL1003) on the infection efficacy of pseudovirus. The viability of hACE2 expressed HEK293 cells in the presence of 3-CQA and HL1003 was shown. Data are presented as the mean ± SEM (n = 3). The IC_75_ of each agent was calculated.

Psuedovirus infection assays were conducted to confirm that 3-CQA's inhibition of spike protein binding reduced infection. 3-CQA was effective in lowering psuedovirus infection for all variants. IC_75_ values for BA.1, BA.2 and BA.4/5, were 256.4, 128.8 and 155.6 μM, respectively. Viability study shown that there was no cytotoxicity effect of 3-CQA on HEK293T-ACE2 cells up to 500 μM (Fig. 5A). Addition of GCEx resulted in the anti-viral infection against ο (BA.1, BA.2 and BA.4/5) psuedoviruses with estimated IC_75_ values equal to 137.1, 50.6, and 139.3 μg/ml, respectively (Fig. 5B). Furthermore, addition of EpEx resulted in the anti-viral infection against ο (BA.1, BA.2 and BA.4/5) psuedoviruses with estimated IC_75_ values equal to 59, 68.5, and 80.9 μg/ml, respectively (Fig. 5C). Finally, addition of RBD antibody (HL1003) resulted in the anti-viral infection against ο (BA.1 and BA.2) psuedoviruses with estimated IC_75_ values equal to 68.5 and 50.4 ng/ml, respectively. The IC_75_ values for the anti-viral effect of HL1003 were 68.5 and 50.4 ng/ml for BA.1 and BA.2, respectively. However, HL1003 was unable to block BA.4/5 the infection of pseudoviruses (Fig. 5D).

**Fig. 6:**
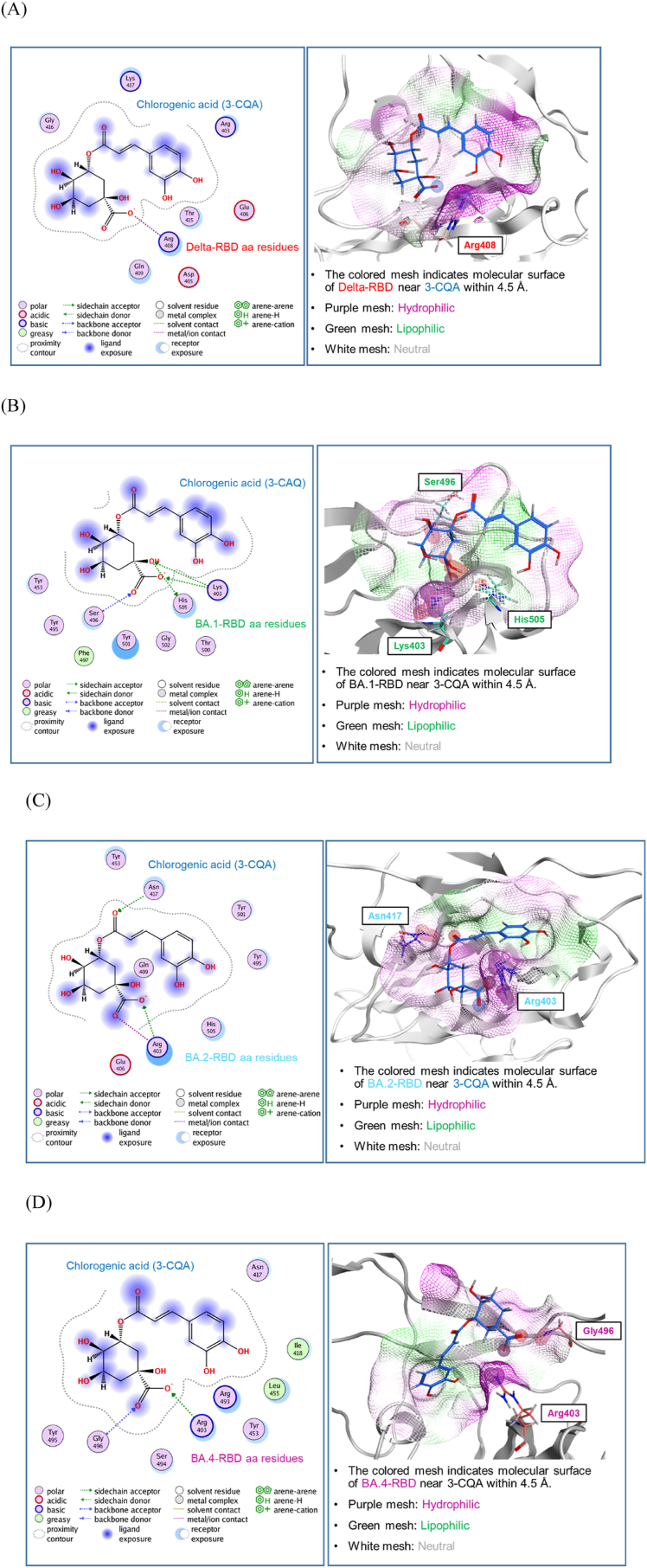
**Docking analysis between 3-CQA and RBD domain of spike proteins from δ and ο variants.** 2D interaction diagram (the left panel) and molecular surface analysis (the right panel) for the interaction of 3-CQA and RBD spike proteins of (A) δ variant, (B) O (BA.1) variant, (C). O (BA.2) variant, (D) O (BA.4/5) variant.

Using crystal structures of the RBD spike protein the docking study of 3-CQA with δ and ο (BA.1, BA.2, and BA.4/5) variants was conducted. In Fig. 6 ligand-protein interaction and the molecular surface properties of RBD and 3-CQA are illustrated. Fig. 6A shows that the carboxylic acid of the quinic acid moiety of 3-CQA interacts Arg 408 of Delta-RBD. On the other hand, 1-hydroxyl group carbonyl group, and carboxylic acid of the quinic acid moiety of 3-CQA mainly interact with Lys 403, Ser 496, and His 505 of BA.1-RBD (Fig. 6B). Furthermore, carboxylic acid and carbonyl group of the quinic acid moiety of 3-CQA interacts with Arg403 and Asn 417 of BA.2-RBD (Fig. 6C). Finally, carboxylic acid and carbonyl group of the quinic acid moiety of 3-CQA interact with Arg403 and Gly496 of BA.4-RBD (Fig. 6D). Molecular surface properties of all RBD near 3-CQA is mainly hydrophilic and lipophilic.

**Fig. 7:**
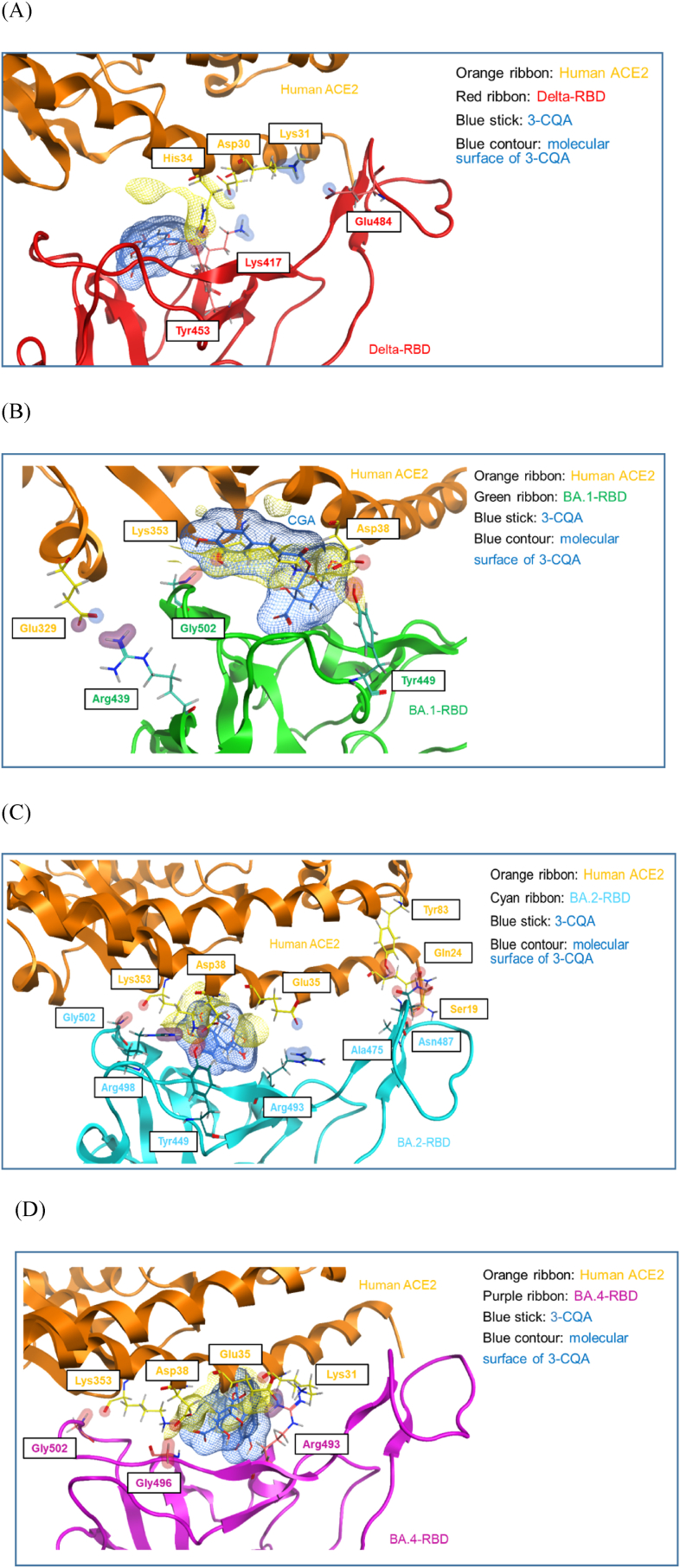
**The proposed superposition of chlorogenic acid with variant RBD spike protein/human ACE2 complex.** The illustration for the superposition of chlorogenic acid with (A) RBD-delta/ACE2 complex, (B) RBD-BA.1/hACE2 complex, (C) RBD-BA.2/hACE2 complex and (D) RBD-BA.4/5 when complexed with hACE2.

Potential steric clash between 3-CQA and RBD spike proteins was further examined by superimposing 3-CQA with RBD spike protein/human ACE2 complex. In Fig. 7A the interactions of amino acid residues between δ-RBD/human ACE2 complex include Lys 417/Asp 30, Glu 484/Lys31, Tyr453/His 34. Superimposing 3-CQA with this complex provided an estimated distance from ACE2 (yellow contours) of around 6 Å, making a steric clash unlikely. In Fig. 7B shows that the interactions within the BA.1-RBD/ACE2 complex involve Arg439/Glu 329, Tyr449/Asp38 and Gly502/Lys353. Superimposition of 3-CQA within this complex leads to an estimated distance of 3 Å from ACE2. Therefore, steric clash between 3-CQA and ACE2 is likely to occur and affects the Tyr449/Asp38 and Gly502/Lys353 interactions.

In terms of the BA.2-RBD/ACE2 complex, Tyr449/Asp38, Ala 475/Ser 19, Asn 487/Gln 24 and Tyr83, Arg493/Glu35, Arg 498/Asp38, and Gly502/Lys353 interactions appear to be important. Simulate distance ACE2 and 3-CQA are ≤3 Å causing steric clash at Gly502/Lys353 (Fig. 7C). Finally, theoretical BA.4-RBD/ACE2 complex was generated by superimposing BA.4-RBD over the BA.2-RBD/ACE2 complex. Proposed interactions within the BA.4-RBD/ACE2 complex include Arg439/Lys31, Arg493/Glu35, Gly496/Asp38 and Gly502/Lys353. Again distances of ≤3 Å between ACE2 and 3-CQA were observed presenting probable steric clashes at Arg493/Glu35 and GLy496/Asp38 (Fig. 7D).

**Fig. 8:**
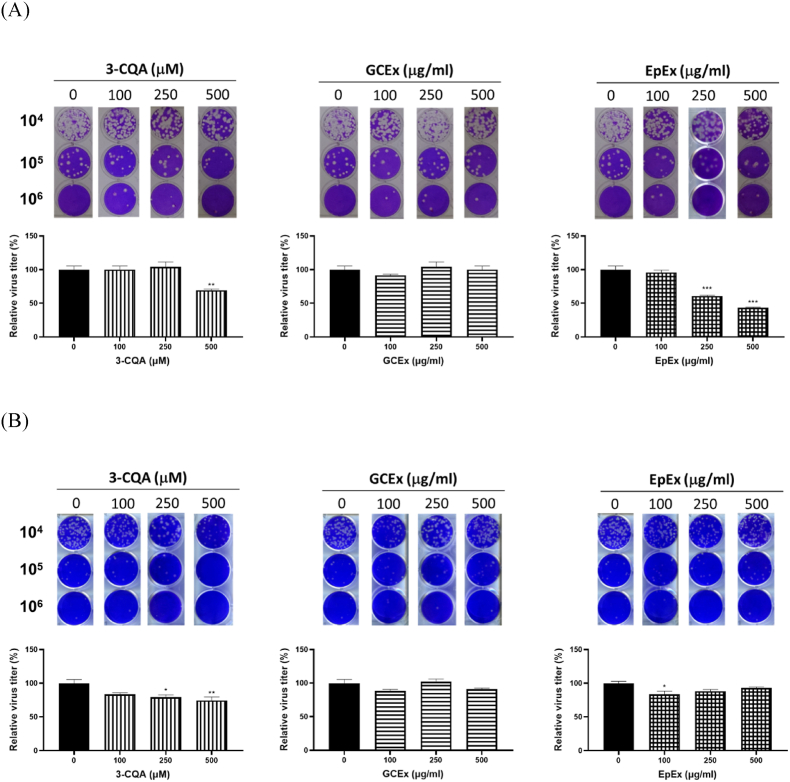
**Anti-influenza activity of EpEX, GCEx, and 3-CQA.** Cells were infected with H1N1 influenza virus for 24 h, with pre-treated (A) or post-treated (B) with 3-CQA, GCEx, or EpEx. The titer of nascent virus of each group was measured by the plaque forming assay, and represented as a representative image and relative virus titer in percentage. Data are presented as the mean ± SEM (n = 3). ∗P < 0.05, ∗∗P < 0.01, and ∗∗∗P < 0.001 compared with Non-treatment group.

Finally, potential anti-influenza effect of 3-CQA and extracts was evaluated. As shown in Fig. 8A, pre-treatment of 3-CQA and EpEx presented anti-influenza activity with 30.5 % and 56.5 % decreases of nascent virus titer at the concentration ranging from 250 to 500 μg/ml. GCEx showed no activity against influenza virus. On the other hand, post-treatment of 3-CQA and EpEX showed modestly activity to reduce influenza virus replication (Fig. 8B).

## 4 Discussion and conclusion

As an RNA virus, SARS-CoV-2 has a high mutation rate, leading to reduced efficacy of both vaccines and neutralizing antibodies against newly emerging variants.^6^^,^^36^ This study demonstrated that a monoclonal antibody targeting the RBD spike protein of the Wuhan reference strain is less effective against Omicron variants. Our data support observations that Omicron variants can weaken COVID-19 vaccine protection.^10^^,^^36^ Reduced efficacy of casirivimab against BA.1 and BA.2, and of imdevimab against BA.1, has also been reported.^37^ In vaccinated persons, the use of dietary phytochemicals capable of blocking spike protein binding could enhance immune defenses against SARS-CoV-2, providing valuable time for the development and clinical validation of next-generation vaccines.

To this end, we investigated the dietary phytochemical 3-CQA, commonly found in foods and herbs.^38^ Previous studies have proposed 3-CQA as a potential therapeutic against SARS-CoV-2. For instance, *in silico* studies have identified multiple SARS-CoV-2 related proteins that may bind 3-CQA, including M^pro^, NSP15, S2, spike ectodomain (active), and ACE2.^23^^,^^24^ However, no experimental evidence existed regarding 3-CQA's inhibitory effect on spike protein binding from VOC.^26^ Our study demonstrates for the first time that 3-CQA blocks spike protein binding to ACE2 in all tested variants, except the Delta variant. Importantly, 3-CQA appears reasonably potent in blocking Omicron variant spike proteins from binding to ACE2. The finding that GCEx and EpEx, which contain 3-CQA, could completely block spike protein binding in current VOC suggests the presence of other derivatives or active components in these extracts.

Molecular docking approaches have been employed to study the interaction of glycosylated spike protein with ACE2 or furin,^39^ as well as to identify anti-SARS-CoV-2 protein targets for small molecules. Given that Omicron variants currently dominate, this study's molecular docking analysis simulated potential steric clashes between 3-CQA and the RBD/ACE2 complex of Omicron variants. Our docking results suggest that the Try 449, Asp38, Lys353, and Glu35 residues of ACE2 are crucial for 3-CQA's blockade of the RBD/ACE2 complex in Omicron variants. Thus, steric hindrance in RBD-ACE2 interactions appears essential for 3-CQA's spike protein binding blockade. Such information could aid future computer-aided drug design and facilitate the identification or synthesis of more potent small-molecule drugs.

In comparing molecular docking results with experimental data, the ELISA-based binding assay results better correlate with molecular docking outcomes, especially when assessing the potential steric clashes in RBD/ACE2 complexes with 3-CQA. Notably, the experimental conditions of the ELISA assay for protein binding are more simplified than those of viral infection assays. SARS-CoV-2 entry mechanisms are complex, involving endosomal (e.g., cathepsin L) and non-endosomal pathways. In non-endosomal pathways, spike protein glycosylation and cleavage by serine proteases (e.g., furin and TMPRSS2) play roles in infection.^40^ Such factors likely account for the observed differences between assay types. Moreover, differences in reporter systems—colorimetric for ELISA versus luciferase for virus assays—can also affect binding assay sensitivity.

Finally, the bioavailability of dietary chlorogenic acids in humans is favorable,^41^^,^^42^ supporting further investigation into the preventive effects of chlorogenic acid consumption against SARS-CoV-2 *in vivo*. In addition, intranasal applications of antiviral agents have been tested in rodents,^43^ suggesting that chlorogenic acids could be formulated as nasal or throat sprays to help curb SARS-CoV-2 transmission. Our preliminary results using an Omicron pseudovirus with GFP reporter genes indicate that this pseudovirus can infect lung and brain tissues expressing hACE2, making this model suitable for evaluating the therapeutic effects of 3-CQA-related extracts against SARS-CoV-2 infection and long-COVID symptoms in the future.

Regarding anti-influenza activity, 3-CQA showed an inhibitory effect on H1N1 viral infection, although its potency was lower than against SARS-CoV-2 variants. Surprisingly, GCEx did not exhibit anti-influenza activity, whereas *Echinacea purpurea* showed convincing effects. Previous studies have shown that *E. purpurea* extract blocks influenza virus binding to cell surface receptors, thus interfering with viral entry.^44^ The Echinaforce Hotdrink, an Echinacea-based drink, was shown to relieve flu symptoms as effectively as oseltamivir in a randomized, double-blind, multicenter clinical trial.^45^ In this study, EpEx pretreatment effectively reduced the titer of H1N1 influenza virus, consistent with previous findings. Overall, our results suggest that 3-CQA serves as a more appropriate biomarker for anti-SARS-CoV-2 activity than for anti-influenza effects.

This study also highlights limitations of 3-CQA as a single phytoconstituent marker. In our estimation, 10 mg/ml of GCEx contains 3-CQA at a molar concentration of 3.67 mM, whereas 50 mg/ml of EpEx contains it at 0.6 mM. Therefore, while 3-CQA and possibly other caffeic acid derivatives are major antiviral components in GCEx, additional active components likely affect binding efficiency in EpEx. Potential candidates include caffeic acid derivatives, bioactive lipophilic alkamides, polyacetylenes, polysaccharides, and glycoproteins, some of which have been suggested to interfere with SARS-CoV-2 pseudovirus and H1N1 infection.^24^^,^^28^ Further investigations are necessary to elucidate these components.

In conclusion, this study experimentally confirms that 3-CQA possesses a broad spectrum of anti-SARS-CoV-2 effects by blocking spike protein binding to ACE2. Dietary supplements rich in 3-CQA could provide similar protective activity against SARS-CoV-2 infection. Thus, 3-CQA could serve as a phytoconstituent biomarker for identifying plant materials with SARS-CoV-2 inhibitory potential.

## Declaration of competing interest

The authors declare that they have no known competing financial interests or personal relationships that could have appeared to influence the work reported in this paper.
