# Supplementary material for: Evaluating the antiviral efficacy and specificity of chlorogenic acid and related herbal extracts against SARS-CoV-2 variants via spike protein binding intervention
Source: J Tradit Complement Med. 2024 Nov 19;15(7):782–93. doi: 10.1016/j.jtcme.2024.11.009 (PMC12624562; doi:10.1016/j.jtcme.2024.11.009)
Supplement: Multimedia component 1 [file mmc1.docx]

**Supplementary Figure 1.**

(A)


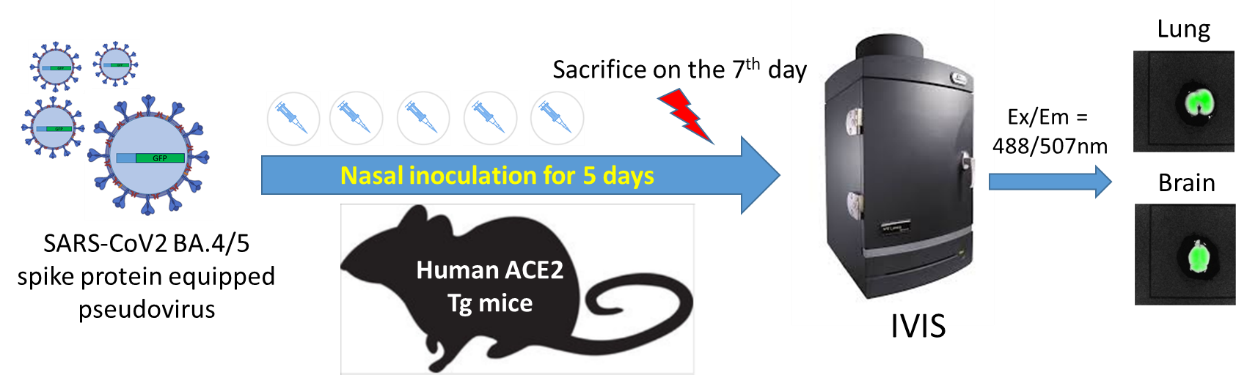


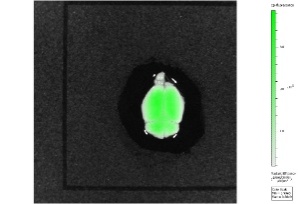

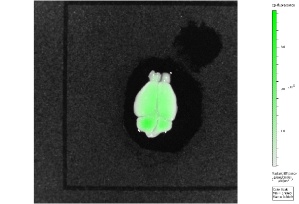

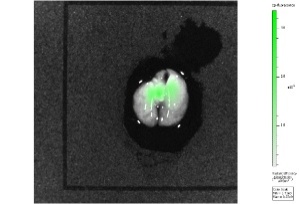
(B) (C)


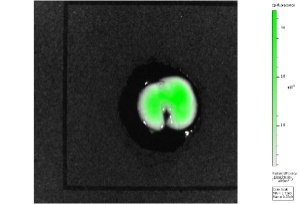


**sFig 1. Nasal inoculation of SARS-CoV2 BA.4/5 spike protein-equipped pseudovirus in human ACE2 transgenic mice results in viral cell entry into lung and brain tissue.** Experimental scheme for establishing a SARS-CoV-2 viral infection mouse model. (B) GFP expression signal detected in lung tissue via IVIS. (C) GFP expression signal detected in brain tissue via IVIS.

**Method**

*Omicron BA.4/5 Pseudovirus Inoculation and GFP Detection*

Eight-week-old B6.Cg-Tg(K18-ACE2)2Prlmn/J mice (hACE2 transgenic mice), kindly provided by Prof. Chiung-Tong Chen from NHRI, were orally administered Oxygolden oxygen tonic at the manufacturer’s recommended volume. The recombinant pseudovirus (Lentivirus) containing the SARS-CoV-2 spike protein (expected Omicron BA.4/5 variant) was thawed and administered nasally (30 μl total volume; viral titer 3039 TU/l) using a dropper to hACE2 transgenic mice. Seven days after the initial inoculation, all mice were sacrificed and systemically perfused. Lungs and brains were collected for GFP expression analysis using the IVIS instrument with Ex/Em=488/507 nm.
